# School Outrages

**Published:** 1876-06

**Authors:** 


### SCHOOL OUTRAGES.

THIRTY years ago a schoolmistress, in a rage, caught hold of the arm of a little girl not in fault, gave it a violent jerk, and with a swing threw her to the other side of the room. To-day, that little girl is a wife, a mother, the accomplished mistress ot a princely mansion, happy in her social position, happy in her husband—who is one of the best of men, but that arm hangs powerless at her side, as it has done from the days of her childhood.

If persons must be screwed up in a coffin, within twenty-four hours of sudden, apparent death, we recommend that a red-hot iron be applied to some portion of the body, which blisters, if there is life, or to cut off a finger, or cut into the flesh several inches.

